# Directional Δ*G* Neural Network (DrΔ*G*-Net): A Modular Neural Network Approach to Binding Free Energy Prediction

**DOI:** 10.1021/acs.jcim.3c02054

**Published:** 2024-03-12

**Authors:** Derek P. Metcalf, Zachary L. Glick, Andrea Bortolato, Andy Jiang, Daniel L. Cheney, C. David Sherrill

**Affiliations:** † Center for Computational Molecular Science and Technology, School of Chemistry and Biochemistry and School of Computational Science and Engineering, Georgia Institute of Technology, Atlanta, Georgia 30332-0400, United States; ‡ Molecular Structure and Design, Bristol-Myers Squibb Company, P.O. Box 5400, Princeton, New Jersey 08543, United States

## Abstract

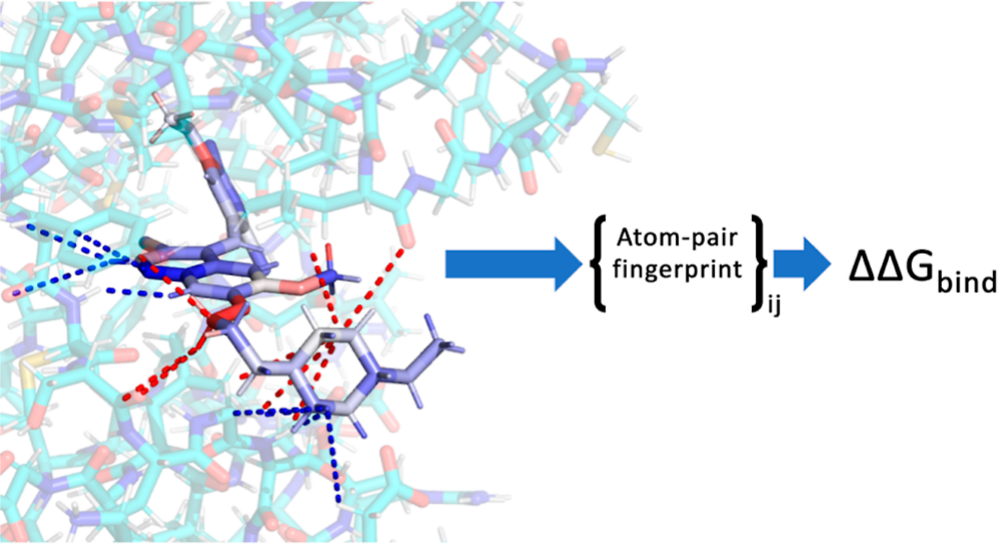

The protein–ligand binding free energy is a central quantity in structure-based computational drug discovery efforts. Although popular alchemical methods provide sound statistical means of computing the binding free energy of a large breadth of systems, they are generally too costly to be applied at the same frequency as end point or ligand-based methods. By contrast, these data-driven approaches are typically fast enough to address thousands of systems but with reduced transferability to unseen systems. We introduce DrΔ*G*-Net (or simply Dragnet), an equivariant graph neural network that can blend ligand-based and protein–ligand data-driven approaches. It is based on a 3D fingerprint representation of the ligand alone and in complex with the protein target. Dragnet is a global scoring function to predict the binding affinity of arbitrary protein–ligand complexes, but can be easily tuned via transfer learning to specific systems or end points, performing similarly to common 2D ligand-based approaches in these tasks. Dragnet is evaluated on a total of 28 validation proteins with a set of congeneric ligands derived from the Binding DB and one custom set extracted from the ChEMBL Database. In general, a handful of experimental binding affinities are sufficient to optimize the scoring function for a particular protein and ligand scaffold. When not available, predictions from physics-based methods such as absolute free energy perturbation can be used for the transfer learning tuning of Dragnet. Furthermore, we use our data to illustrate the present limitations of data-driven modeling of binding free energy predictions.

## Introduction

The drug discovery process, historically reliant on the discovery of active compounds from natural sources or fortuity, was reshaped by the ability to synthesize large numbers of proteins that are implicated in diseases. The process of screening many possible small molecule drug candidates against these proteins is known as reverse pharmacology or target-based drug discovery and is the basis of modern small molecule drug discovery efforts.^1,2^ However, ideating, synthesizing, and screening a massive number of compounds remains a costly endeavor.^3^ Automated in silico prediction of safe and effective drugs is, therefore, among the most alluring goals of the computational sciences.

Developments in quantum chemistry and statistical physics have introduced theoretically rigorous mathematical instruments to deduce some properties associated with drug efficacy. However, the scale and complexity of biology preclude exact or near-exact treatment of the most salient properties, such as binding constants, kinetic on- and off-rates, and solubilities. Approximate computational treatments enjoy broad industrial application, especially binding constant estimates based on simulation via molecular dynamics (MD) and data-driven approaches.^4,5^

The free energy of binding of a drug molecule to a target protein is among the most important indicators of the efficacy of the prospective drug. Statistical mechanics allows estimation of binding free energies, most frequently via “alchemical” approaches like free energy perturbation (FEP), which usually computes relative binding free energies between similar ligands.^6^ Considerable effort has been made to compute *absolute* binding free energies, which avoids the similar-ligand stipulation but is practically much more challenging.^7^ These techniques require molecular mechanics force field parametrization since ab initio forces are generally too costly for molecular dynamics simulations. FEP can be applied to any protein–ligand system so long as reasonable force field parameters are available or can be deduced for the ligand of interest. Unfortunately, the requisite molecular dynamics trajectories for FEP are themselves costly, and presently limit its industrial application to tens or hundreds of ligands for a given project. The requirement that ligands be similar in relative free energy computations is an additional deficit that usually requires additional intermediates or reference ligands to overcome. Despite these challenges, relative and absolute binding free energy prediction is becoming more routine in light of improved force fields, computing power, and best practices in their execution.^8,9^ Site identification by ligand competitive saturation (SILCS) provides an alternative MD-based approach to approximating the binding free energy by creating binding probability maps with diverse small molecule fragments and employing a similarity-based atomic-additive ansatz for the binding free energy of new ligands.^10,11^

End point methods attempt to reduce the binding free energy, which is inherently a property of a thermodynamic ensemble, to a property of a very small number of configurations, namely, the bound pose of the ligand with the target protein, and protein and ligand each unbound in solution. The bound pose could be experimentally obtained with X-ray crystallography, but it generally is not known a priori and is frequently approximated with molecular docking techniques. Popular forms like molecular mechanics generalized Born surface area (MM-GBSA)^12^ and molecular mechanics Poisson–Boltzmann surface area (MM-PBSA) attempt to partition the binding free energy into physically motivated terms that approximate the ensemble property.^13^ Specifically, both express the free energy as a difference in states
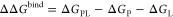
1with free energies *G* and P denoting a protein, L, a ligand, and PL the complex.^14,15^

In contrast to end point and alchemical approaches, ligand-based methods approximate binding free energies (or related quantities) with a data-driven statistical algorithm that has access only to the ligand molecular structure, which is represented as a collection of atoms and bonds.^14,16^ There exists a large variety of ways to featurize molecular graphs, including but not limited to traditional fingerprinting techniques^17^ and those based on neural network graph convolution.^18^ Since ligand-based approaches have no access to protein information, they are parametrized and useful only for individual protein targets and therefore require data and reparameterization for each new target. Information about the three-dimensional bound ligand pose can be foregone because some ligand ensemble information (such as flexibility, which impacts entropy) is implicitly encoded in the molecular graph, and the molecules in the training set are chosen to share binding motifs with those in the prospective “test” set. Therefore, a sort of focused overfitting yields strong predictive results, even in the absence of physically motivated functional forms. These models generally operate only over a limited chemical space in the specific model applicability domain, so they are expected to degrade in performance on chemically dissimilar molecules to the training set.^19^ Despite these stipulations, ligand-based approaches remain an accurate choice when appropriate training data are available.

Molecular docking is a technique used to elucidate the correct bound orientation or “pose” of a ligand in a protein binding pocket. This procedure requires a scoring function to determine the correctness of a pose. These functions differ from both the ligand-based and alchemical approaches above, as they must address arbitrary protein–ligand pairs, be very computationally inexpensive, and operate on three-dimensional coordinates of complexes. Unfortunately, scoring is the weakest step in docking methodologies. In fact, in the majority of the cases, it is unable to rank-order even a list of prospective ligands (or a hit list). Common scoring functions used in molecular docking software dramatically simplify the thermodynamics of the binding event. Scoring functions can be grouped in three families: molecular mechanics force fields, empirical, and knowledge-based.^20^ In molecular mechanics, the energy expression includes intramolecular and intermolecular contributions. Empirical scoring functions approximate the binding energy as a sum of the uncorrelated energy terms. Coefficients are obtained from regression analysis of a set of ligands with known experimental binding energy to the target and with available X-ray structures of the complex. Knowledge-based scoring functions are composed of multiple weighted molecular features related to ligand–receptor binding modes. The features are often not only atom–atom distances between protein and ligand in the complex but also the number of intermolecular hydrogen bonds or atom–atom contact energies. A large number of X-ray diffraction crystals of protein–ligand complexes are used as a knowledge base. A putative protein–ligand complex can be assessed on the basis of how similar its features are to those in the knowledge base. These contributions are summed over all pairs of atoms in the complex, and the resulting score is converted into a pseudoenergy function estimating the binding affinity. The coefficients of the features can be fitted using linear regression analysis, but other nonlinear statistical approaches can also be used, such as a neural network, Bayesian modeling, or other machine learning techniques such as random forests. Examples include DrugScore^21^ and RF-Score.^22^ Disadvantages of knowledge-based scoring functions are difficulties in the evaluation of the chemical–physical meaning of the score and the risk of errors when trying to predict ligands not included in the training set.

Neural networks are a popular type of nonlinear regression model for inferring molecular properties after being trained on examples of molecules and some known target property. Molecules can be represented as graphs with nodes at atomic nuclei and edges between nuclei.^23^ Edges usually encode some proximity information, such as known chemical bonding or Euclidean distances. Many existing graph neural network (GNN) models operate on two-dimensional molecular graphs, which only includes molecular topology as described in the previous section.^18,24,25^ Many architectures attempt to utilize GNNs and related neural network and classical approaches to characterize binding affinities and docking poses.^26−32^

Other GNN approaches leverage 3-dimensional information by including explicit distance information along graph edges.^14,33^ In this work, we describe a GNN that operates on the full 3-dimensional coordinates of a protein–ligand complex. This type of GNN is frequently used for predicting single-point molecular properties such as ground-state energies, and a large number of architectures have emerged for these tasks.^34−38^

Since only distances are encoded along edges and not vector-valued displacements, the output of these networks is inherently invariant to rotations and translations of the molecule. Invariant outputs are generally a benefit, as properties like energy are similarly invariant. However, vector properties like force on atomic nuclei are *equivariant* with respect to molecular rotation; that is, they rotate as the molecule rotates. Although forces can be obtained by differentiating an invariant network with respect to nuclear coordinates, it has been shown that architectures imbued with equivariance are drastically more data efficient and accurate for these tasks.^36,37,39^ These models accommodate vector- and tensor-valued features on nodes and edges that can iteratively update just as scalar features do in conventional GNNs. Surprisingly, equivariant models even show improved data efficiency when predicting *invariant* properties. It is, therefore, evident that equivariance is a natural bias to encode into models for molecular property prediction.

Here, we introduce an equivariant GNN with tensorial features that predicts the binding affinity of arbitrary protein–ligand complexes. This model incorporates ligand pose information as well as specific protein–ligand contacts in three dimensions. The model trained for arbitrary complexes can be readily tuned to a specific protein by continuing to train with a small number of reference data.

## Methods

### Neural Network Architecture

**Figure 1:**
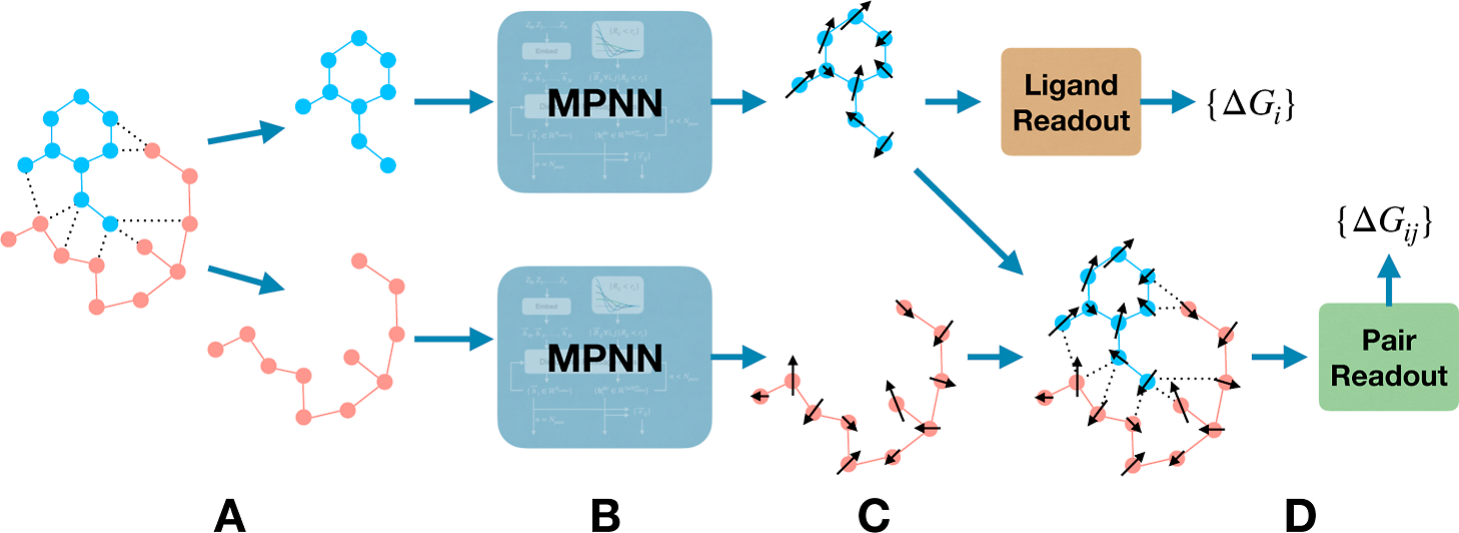
(A) The raw input is a protein–ligand system with edges defined by a distance criterion. (B) The protein and ligand are separately featurized with a directional message-passing neural network depicted in Figure 2. (C) Each atom in each system has a set of scalar features  and a set of vector-valued features , represented by arrows. (D) Predictions of free energy contributions are read out according to eqs 3 and 4 and combined by eq 2, where *i* denotes ligand atoms and *j* denotes protein atoms.

**Figure 2:**
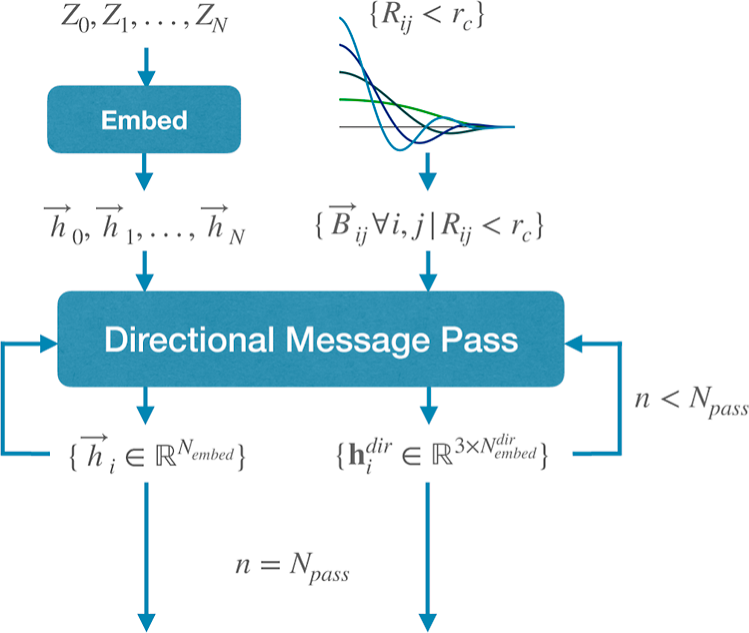
Schematic representation of the message passing phase depicted in Figure 1B, mapping from the set of element identities *Z*_*i*_ and interatomic distances *R*_*ij*_ to scalar and directional atomic features,  and **h**_*i*_^dir^, respectively.

The core of the Dragnet architecture is an equivariant GNN operating over both the protein and ligand, which are treated as graphs embedded in a three-dimensional space. We specifically use a message-passing neural network, a type of GNN that iteratively confers information along graph edges in order to generate useful latent representations of nodes and/or edges. The message-passing unit is based on the E(n) equivariant GNN (EGNN) of Satorras et al.^36^ Here, we reiterate architectural details of the EGNN and introduce aspects unique to this application. A schematic depiction of the architecture is shown in Figures 1 and 2. Nodes are placed on nuclear coordinates from either crystallographic data or molecular docking, and edges connect atoms within the same molecule (protein or ligand) within a specified cutoff radius. Dragnet is, therefore, an example of a neural network end point method, as other members of the thermodynamic protein–ligand ensemble are not explicitly included in the representation. Directional messages are conferred along the two disconnected graphs and update the node states, which have invariant (scalar) and equivariant (tensorial) features. Boldface notation indicates tensorial quantities, such as the set of vector-valued node features ascribed to each atom **h**_*i*_^dir^. The only explicit inputs to the model are 3-dimensional coordinates of all atoms, atomic identities, and whether each atom belongs to a protein or ligand. Interatomic distances are computed and represented on a small orthogonal radial basis of Bessel functions. Atomic identities are embedded in a continuous vector by a small dense neural network. Ownership of each atom by the protein or ligand is represented implicitly by graph formation and through the energy readout, as described below.

#### Free Energy Readout

A “readout” function transforms the hidden states into a system-wide prediction.^23^ Many efforts to infer potential energies use neural networks that operate on node hidden states to yield an atomic contribution to a total energy.^23,34−38,40−45^ This has no physical grounding but satisfies property extensivity and serves as an appropriate inductive bias for chemical systems, exploiting the locality.

In this work, the readout function is a combination of a sum over free energy contributions from *atoms* in the ligand and *atom-pairs* of proximal ligand and protein atoms
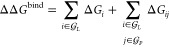
2where  and  denote the ligand and protein (or protein pocket) graphs, respectively. The rationale for atomic readouts is 2-fold: Most generally, atomic readouts over protein or protein-pocket atoms would carry analogous information, but we find that these inferences yield no performance improvements and are challenging to tune. Atomic readouts in this work take the form
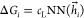
3with the set of scalar node features given by  and a scaling constant *c*_L_. NN is a small dense feed-forward neural network with weights shared across all atom readouts. The pair readouts have a similar form, but take additional features that are derived from interatomic distances and simple manipulations of directional atomic features
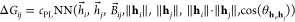
4with the distance *r*_*ij*_ projected onto a set of Bessel functions to yield , magnitudes of directional features , the set of *n*_f_ scalar products between corresponding feature indices, and the cosine of the angle between each pair of directional features . We forego symmetrizing this expression with respect to permuting ligand atoms *i* and protein atoms *j* and enforce this ordering to maximize function flexibility. The choice of using pair readouts is inspired by the recent AP-Net project in this group for predicting intermolecular interaction energies, where it was discovered that inferring over atom-pairs is a far more appropriate bias when concerned with noncovalent interactions.^41,42^ Improvements were especially apparent in strong directional interactions such as hydrogen bonding, which are nearly universal in protein–ligand interactions. Therefore, we expect the same architectural choice will help in the adjacent *free* energy problem. The software for the current project relies upon some of the infrastructure developed for our most recent version of AP-Net.^46,47^

1. Empirically, ligand-based modeling is impressively performant, so it would be useful to adapt ligand-based frameworks to the Cartesian data.
2. Quantities like strain and entropy are not included elsewhere and the signal relating to those contributions is partly contained in the ligand geometry.

All readout functions used in this work regress toward p*K*_*i*_, the negative logarithm of the protein–ligand dissociation constant, not the free energy of binding Δ*G*_bind_. This is for convenience since existing data sets report p*K*_*i*_ and the two quantities are linearly related at a given temperature, so are identical in the context of regression. System predictions are shifted by a single, trainable parameter initialized to the mean p*K*_*i*_ of the training set, which drastically improves the training convergence rate. With this shift parameter, atomic and atomic-pair readouts represent deviations from the average training set binder. As such, analysis of predictions at the atomic and atom-pair levels should be performed relative to other atoms in the same system or systems that share a protein target, not in absolute terms. This is consistent with typical drug discovery workflows. Predictions on whole systems (that is, the sum of atomic and atom-pair contributions) are approximations of p*K*_*i*_ and can be interpreted as such.

To explore the limits of data-driven end point free energy prediction, we additionally trained models with an alternative readout modality: A ligand-only readout neural network infers the free energy as a sum of contributions from atoms in the ligand. Since the protein and ligand graphs (from which atomic features are generated) are disconnected, this readout has no access to explicit protein geometry. This is reminiscent of ligand-based 2D topology approaches, but here the network is afforded a 3-dimensional crystal structure or docked configuration of the ligand.^48^

#### Computational Complexity

The most costly step in the various Dragnet architectures is the message-passing phase, requiring edgewise enumeration of messages for each pass. Without cutoffs, this scales like (N_protein_^2^) since N_protein_ ≫ N_ligand_. Importantly, appropriate cutoffs effectively transform the message-passing phase and the atom-pair readout from (N^2^) complexity to (N) at the system sizes relevant to this work. Indeed, with these sparse evaluations, the cost of inference is less than the requisite docking. Performance converges around cutoffs of 5 Å for both readout and energy functions. Ligand-only readout approaches eliminate the need to parameterize the protein, which is the most time-consuming step. Therefore, ligand-only networks are generally 3–5 times faster to train and infer than full protein–ligand networks.

### Data

For further discussion, the term “global” is used to denote a protein–ligand data set that contains a large number of crystal structures of different proteins complexed with ligands with known binding affinity. By contrast, “local” will denote data sets containing a single protein target and a diverse set of related ligands with known activity for that particular system. For these experiments, we have access to absolute free energy labels (or proxies thereof), so we predict those, although relative free energies between ligands could also be predicted. Some experiments operate on a union of local data sets.

The global training set chosen for this work is the 2018 PDBbind refined set with members of CASF2016 removed.^49,50^ This training set contains 4024 protein–ligand complexes and will hereafter be referred to as PDBbind or as the global set. The 285 members of CASF2016 are reserved as a test set, as has become a frequent practice for global scoring functions.^51^ To isolate CASF2016, 5-fold cross-validation and hyperparameter selection are performed only over PDBbind.

The local data sets include 27 sets from Binding DB^52^ (http://www.bindingdb.org/validation_sets/index.jsp). Each set includes congeneric small molecules with a range of affinities for a single protein target, and at least one complex in each series has a structure in the PDB, as a basis for modeling the rest of the series. They include in total 14 different protein targets. In addition, one set (referred to as Set 0) was created using data available from ChEMBL Database^53^ for known agonists of the S1P_1_ receptor. A summary of the different local data sets is included in Table 1.

**Table 1:** Summary Information for Local Datasets Extracted from BindingDB and ChEMBL

set #	target	PDB ID_ligand ID	number of ligands	train poses	test poses
0	S1P1R	3V2Y_ML5	1215	1352	340
77	BACE1	3IN3_472	313	266	65
78	BACE1	2IQG_F2I	762	835	191
87	BACE1	4WY1_3VO	190	173	51
381	CDK2	2A4L_RRC	176	141	35
384	CDK2	2C5V_CK4	87	63	16
484	MDM2	2LZG_13Q	187	150	40
488	MDM2	3LBL_MI6	359	329	81
505	EGFR	1M17_AQ4	514	498	150
677	IRAK4	4ZTL_4S1	509	520	134
707	HLE	5A09_JJD	822	1376	321
761	MAPK1	4XJ0_41B	82	95	24
762	MAP1	2OJG_19A	348	291	71
786	MAPK14	1KV1_BMU	426	333	84
787	MAPK14	1KV2_B96	401	315	78
789	MAPK14	4FA2_SB0	106	92	23
796	MAPK14	2RG5_279	127	98	25
889	PPAR	1FM9_570	304	250	62
894	PPAR	3KMG_538	335	290	72
993	QR1	1RNE_C60	452	324	83
1004	QR1	3O9L_LPN	92	73	19
1076	PIM	5DIA_5E6	315	291	73
1081	PIM	5EOL_5QO	342	586	158
1163	TNK2	4ID7_1G0	333	267	67
1164	TNK2	3EQR_T74	30	25	6
1170	BTK	5FBN_5WF	1186	1166	299
1171	BTK	3OCS_746	139	141	31
1209	SYK	3FQE_P5C	656	543	135

#### Preparation of Binding DB and ChEMBL Sets

All protein–ligand complexes were prepared with an automatic Python pipeline (called Mario) based on the Schrödinger Suite 2021-2.^54^ The workflow uses standard options to execute the following steps: (1) convert the activity to p*K*_*i*_ and divide the ligands into training (80%) and test sets (20%) using stratified sampling based on their reported activity; (2) prepare the protein (using the Protein Preparation Wizard, Schrödinger);^12,55,56^ (3) prepare the ligands (using LigPrep, Schrödinger); (4) generate the Glide grid for docking;^57^ (5) dock the ligands with Glide; (6) rescore the poses with MM-GBSA. Default options were generally used, but for Ligprep a pH = 7.4 and a pH threshold of 0.1 was applied. The standard precision (SP) mode was selected for Glide docking, and the crystal structure ligand was used as a shape-based constraint, a constraint to predefine the accessible internal ligand geometry. This resembles a typical drug-discovery workflow, where the crystal structure of a homologous compound is known. Top-scoring poses for ligands were used as inputs for Dragnet. Results from machine learning models are the average of five models from 5-fold cross validation. The test set is always held out for the final evaluation.

#### Online Data Augmentation

Since Dragnet is rigorously equivariant, data augmentation in the form of rotations and translations is strictly unnecessary. Instead, we employ small random perturbations of the atomic Cartesian coordinates during training. We have previously used this technique to address overfitting problems in predicting interaction energies of crystal structures after training on artificially generated structures with constant intramolecular coordinates.^42^ We expect this augmentation to regularize against overfitting to the specific docked or crystallographic pose within a bound state and retain the same free energy label. This augmentation also incorporates the uncertainty inherent to docking and crystal structure coordinates. Coordinates are augmented randomly within a 0.1 Å sphere each time an example is used for training; augmentation is turned off during inference.

#### Absolute FEP Calculations

We evaluated the possibility of replacing experimental data with computed binding affinities from absolute FEP (AFEP) to be used for the transfer learning optimization of Dragnet. The test involved 6 related agonists of the S1P_1_ receptor: CHEMBL3908375, CHEMBL1080880, CHEMBL1081654, CHEMBL3896108, CHEMBL3982822, and CHEMBL3931249. The protein coordinates were downloaded from the Protein Data Bank (PDB ID 3V2Y).^58^ Using Maestro (Schrödinger) Prime,^59^ the fusion protein T4-lysozyme was removed, missing loops were added, and thermostabilizing mutations were back mutated to corresponding wild-type human amino acids. The protein was refined with the Protein Preparation Wizard. CHEMBL3908375 was docked using Glide SP using as a shape-based constraint for the crystallized ligand, (*R*)-3-amino-(3-hexylphenylamino)-4-oxobutylphosphonic acid (ML056).^60^

The resulting S1P_1_ Receptor in complex with CHEMBL3908375 was prepared for molecular dynamics (MD) simulation using the System Builder in Maestro. The protocol embedded the complex in a POPC (1-palmitoyl-2-oleoyl-*sn*-glycero-3-phosphocholine) membrane bilayer, added equilibrated water molecules in the simulation box, and neutralized the total charge with the correct type and number of ions. The system was equilibrated using Desmond MD software 2022-2^61^ using the default protocol for membrane systems plus an additional 50 ns MD simulation (*NVT* ensemble at 300 K temperature). CHEMBL3908375 was used as shape restraint to dock the other 5 ligands in the equilibrated system using Glide SP. The absolute free energy of binding of the 6 ligands was evaluated using the AFEP (AFEP+) method in Maestro 2022-2 with default settings.^62^

### Training

#### Direct Learning

In this work, “direct” learning refers to training a neural network with randomly initialized weights on a data set of protein–ligand systems and corresponding binding free energy labels. Direct models are trained on the global PDBBind data set as well as on each individual local, protein-specific data set. Hyperparameters determined from cross-validation on the global set are used for all subsequent direct models. We note that reoptimizing hyperparameters for local sets remains an open research direction since local sets usually lack the data quantity required for rigorous validation. Network quality may be sensitive to the hyperparameter choice in this adjacent domain.

#### Transfer Learning

Machine-learned scoring functions operating on two-dimensional molecular representations of the ligand are very successful when trained on small data sets of congeneric molecules. By contrast, neural networks excel when large amounts of labeled data are available. Transfer learning attempts to bridge the disparity in data efficiency by starting from a model pretrained on some related task. For this work, a Dragnet model is first pretrained on the global PDBBind data set and then used as a base model from which a local, protein-specific model is trained. A similar procedure was attempted for the binding affinity task by De Fabritis et al. using a convolutional network and attempting to learn differences in binding free energies by reading out a difference in hidden states between two poses.^63^ We instead readout the hidden states produced by equivariant message passing, requiring no rotational data augmentation, and we infer absolute binding free energies. Although it is common in transfer learning to constrain early layers of neural networks when training on the secondary data set, we found this to be detrimental to performance and instead leave all weights trainable. This is, therefore, equivalent to initializing with a reasonable model and tuning its weight for the new task. Weight momenta from the original training run are not retained, and the transfer learning procedure is made robust with respect to large gradients by utilizing the same warm-up procedure described below and in the Supporting Information for other networks.

#### Hyperparameter Tuning

In a nonexhaustive search, we found accuracy to be either convergent (plateauing at large values) or insensitive to hyperparameters like the depth and width of all dense neural networks, the number of message passes, activation functions, and embedding dimensions. Therefore, we chose the first convergent value for those hyperparameters, which are listed in the Supporting Information. However, accuracy was sensitive to learning rate, dropout between dense layers, batch normalization, learning rate warmups, and online augmentation magnitudes. For these, we conducted a random search over a large range of plausible values and trained a neural network on the PDBBind global data set with each parameter set. The set of hyperparameters producing the lowest validation error was used to create a final 5-member ensemble and one such model as the basis for transfer learning experiments.

## Results and Discussion

### Global Models

**Figure 3:**
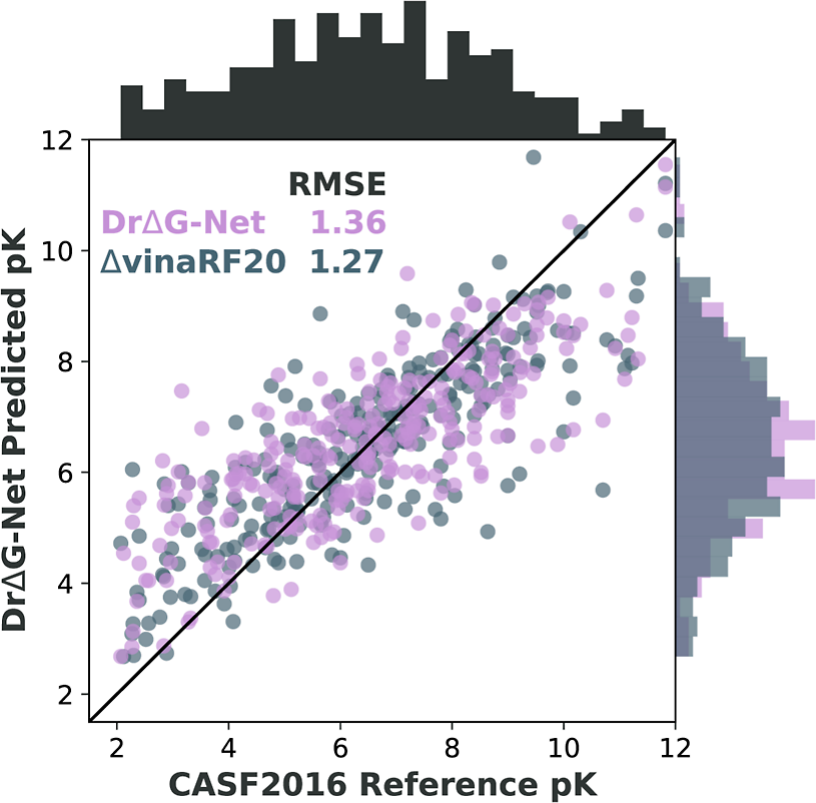
A comparison between the global Dragnet and the ΔVina-RF20 scoring function on the CASF2016 test set. Data distributions of both models and of the reference values are shown on the right and top spines, respectively.

Figure 3 illustrates the global Dragnet model accuracy compared to the popular random forest model Δ_vina_RF20.^64^ Δ_vina_RF20 is a correction to the Vina docking score;^65^ the correction explicitly encodes many intuitive chemical features such as hydrophobicity and rotor counts. Dragnet achieves an RMSE of 1.36 p*K* units, slightly higher than the 1.27 of Δ_vina_RF20. Interestingly, the two models have very similar out-of-sample performances despite having qualitatively different functional forms and featurization techniques. The two share a pervasive characteristic of data-driven free energy models: they artificially compress the output distribution to reduce the frequency of extreme values. The physical priors available to Δ_vina_RF20 do not appear to improve predictions of very strong and very weak binders, resulting in a similar density profile to Dragnet on the right spine of Figure 3. It has previously been noted that the performance of Δ_vina_RF20 on this test set may benefit from some data leakage from parametrizing the underlying Vina method on the test set.^15,51^

Surprisingly, a Dragnet network trained only on bound ligand geometries (in the absence of any explicit protein atoms) attains *nearly the same accuracy* (RMSE of 1.43 p*K* units) as the Dragnet model trained on full protein–ligand complex geometries. This experiment is similar to that performed in the work of Volkov et al.,^14^ with the exception that Dragnet utilizes directional message passing and has more permissive distance-based criteria for enumerating neighbor lists (edges between proximal atoms). Both modifications are known to significantly improve accuracy in the related problem of inferring potential energies,^36,37,39^ but do not impact the memorization behavior that makes ligand-only networks similar in accuracy to protein–ligand networks. These findings reinforce the conclusions of Volkov et al.

Most current data-driven free energy predictions exhibit very similar errors to the ones presented here.^15^ Several years of architectural modifications have improved free energy prediction by only small percentages on benchmark data sets.^66−68^ This is unlike the related task of potential energy prediction, where recent innovations have resulted in profoundly improved accuracy and transferability.^36,37,39^ There exist several key differences between the two targets:

1. Potential energy prediction data is noiseless, as it is deterministically produced by a quantum mechanical method. Experimental free energies include measurement noise and are sometimes derived from proxies of the true binding free energy (e.g., IC_50_).
2. Potential energies are functions of single static configurations, so mapping a configuration to its energy with a machine-learning method is a reasonable approach. Predicting free energy from a single pose neglects all other configurations, including the unbound configuration, that appear in known expressions for computing absolute binding free energies. This includes entropic effects, which themselves represent an immense challenge.
3. Benchmark potential energy data sets are generally much larger than free energy data sets, as they can be computed more easily.

We therefore expect further advancements in the field of predicting the binding free energy of arbitrary protein–ligand complexes with data-driven approaches to address the differences described above. Incremental architectural improvements without improvements to the data sets or system representation may never realize the gains seen in the field of potential energy prediction.

### Local Models

Fast global scoring functions are usually unable to accurately rank the binding affinity of related ligands for a specific protein target. Likewise, docking scoring functions are generally not highly correlated to the ligand activity. The local sets included in this study are especially complex, since the binding modes of the ligands are unknown. Mispredictions can be caused by the wrong ligand binding modes, errors in the scoring function, or both. When multiple ionic states, tautomers, and enantiomers are possible for one ligand, all forms are docked, and the best scoring pose is used. These local data sets represent realistic cases in drug discovery projects in the hit-to-lead or lead optimization stages. In these settings, experimental data are sometimes available for structurally related ligands, and the experimental binding mode of a representative compound is available from X-ray crystallography. The objective is to improve the activity of the chemical series by taking advantage of all available data. For this particular task, a global scoring function is not optimal: as shown in Table 2 the average r^2^ of Glide^69^ and MM-GBSA^13^ is 0.14 and 0.11, respectively. Similar poor performance is obtained with the global version of Dragnet with an average r^2^ of 0.17: despite the strong performance on the CASF2016 test set, it lacks the fidelity to distinguish ligands with differences in binding strength of less than a p*K* unit. It is important to note that this does not appear to be an example of overfitting as the MUEs observed are often comparable to those seen in CASF2016. Rather, this is a consequence of the ligand series in these sets occupying a very small range of p*K* values, which is consistent with typical discovery projects.

**Table 2:** Predictive Performance on the Holdout Set of Glide Docking Scoring Function, MM-GBSA, Global and Local Versions of Dragnet, and GBM^a^

set #	target	glide	MM-GBSA	global Dragnet		local Dragnet		GBM	
		*r*^2^	*r*^2^	*r*^2^	MUE	*r*^2^	MUE	*r*^2^	MUE
0	S1P1R	0.00	0.03	0.18	1.13	0.53	0.77	**0.75**	0.59
77	BACE1	0.18	0.09	0.27	0.91	0.41	0.75	**0.79**	0.37
78	BACE1	0.00	0.05	0.07	1.72	**0.74**	0.16	0.14	0.76
87	BACE1	0.22	0.08	0.20	0.92	**0.50**	0.63	0.09	0.79
381	CDK2	0.09	0.05	0.13	1.30	0.59	0.66	**0.72**	0.57
384	CDK2	0.62	0.23	0.12	1.43	**0.73**	0.19	0.52	0.59
484	MDM2	0.12	0.14	0.53	0.89	0.57	0.8	**0.59**	0.68
488	MDM2	0.13	0.22	0.00	1.49	**0.55**	0.51	0.43	0.60
505	EGFR	0.07	0.13	0.07	1.14	0.53	0.65	**0.64**	0.51
677	IRAK4	0.42	0.3	0.28	1.22	0.53	0.73	**0.62**	0.60
707	HLE	0.05	0.06	0.10	1.26	0.32	0.42	**0.40**	0.30
761	MAPK1	0.14	0.27	0.28	0.60	**0.46**	0.24	0.12	0.52
762	MAP1	0.00	0.01	0.10	1.10	0.52	0.61	**0.56**	0.57
786	MAPK14	0.04	0.03	0.13	0.93	0.39	0.7	**0.67**	0.38
787	MAPK14	0.09	0.04	0.01	1.49	0.41	0.66	**0.60**	0.43
789	MAPK14	0.36	0.2	0.38	1.76	**0.58**	0.39	0.57	0.46
796	MAPK14	0.00	0.00	0.12	0.99	**0.57**	0.26	0.32	0.54
889	PPAR	0.00	0.06	0.11	1.80	**0.73**	0.52	0.57	0.70
894	PPAR	0.14	0.03	0.02	1.61	**0.56**	0.32	0.35	0.59
993	QR1	0.02	0.04	0.08	0.91	**0.73**	0.39	0.42	0.68
1004	QR1	0.29	0.35	0.07	0.78	0.46	0.57	**0.69**	0.41
1076	PIM	0.20	0.02	0.29	1.83	0.37	0.79	**0.73**	0.41
1081	PIM	0.00	0.04	0.01	0.79	0.45	0.58	**0.54**	0.49
1163	TNK2	0.00	0.00	0.04	1.83	**0.87**	0.16	0.12	0.87
1164	TNK2	0.59	0.67	**0.86**	2.22	0.58	0.33	0.17	0.50
1170	BTK	0.04	0.04	0.01	1.55	**0.62**	0.33	0.41	0.57
1171	BTK	0.15	0.12	0.19	1.39	**0.50**	0.53	0.46	0.69
1209	SYK	0.00	0.00	0.06	1.61	**0.40**	0.47	0.31	0.46

a MUEs are given in units of p*K*_*i*_. Boldface denotes the model with the highest *r*^2^ value.

For binding prediction tasks with some small amount of data available, it is generally more effective to create a custom scoring function optimal for the particular target and chemotype under investigation. The API included in Dragnet allows easy scoring function training using a set of available ligand docked poses with known activity. For this purpose, every set from Binding DB and the ChEMBL set was prepared with the Mario pipeline. For every set, 80% of the ligands were used as training compounds, with the remaining 20% reserved as test compounds. The division was derived on stratified sampling based on the compound activity to obtain a similar target distribution between the training and test sets. After protein and ligand preparation, compounds were docked in the target binding site using the reference crystallized compound as a shape-based constraint to guide the conformational search. Final poses were used as inputs for the local Dragnet training.

**Figure 4:**
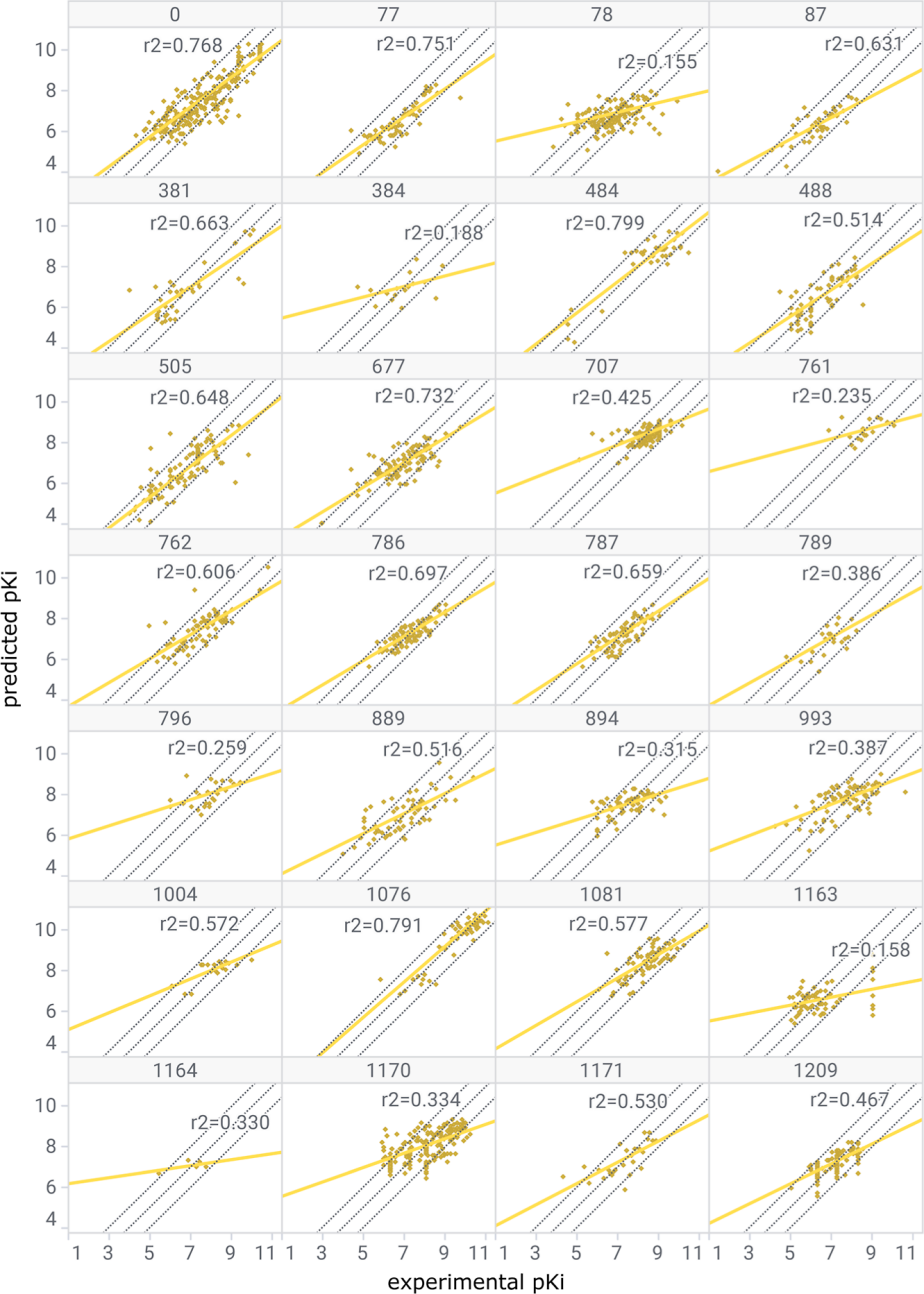
Experimental p*K*_*i*_ (*x*-axis) versus Dragnet predicted p*K*_*i*_ (*y*-axis) for all test sets, each consisting of 20% of ligands excluded from local Dragnet training and cross-validation. The set number is included at the top of every plot. Correlation factor *r*^2^ is reported for every set. Set 0 is based on ChEMBL data, while the other 27 are based on Binding DB. The line of best fit for each correlation plot is illustrated in yellow.

As expected, the resulting local scoring functions outperform the global methods (average *r*^2^ = 0.5 ± 0.2, Figure 4). For all sets, the mean unsigned error (MUE) was always below 1 p*K* units with an average of 0.54 ± 0.13 p*K* units (Table 2). These local models performed comparably to ligand-based methods like gradient boosted machines (GBM) operating on Morgan fingerprints of each ligand, which achieved an average absolute error of 0.55 ± 0.14 p*K* units across all sets.^70,71^

The quality of the local methods is far better than the best general Dragnet and end point models available as they have access to some reference binding affinities for the given target and congeneric ligands. All local models are trained on docked poses, not crystallographic poses, so little quality degradation is expected when moving to real-world use-cases where poses may be generated by docking, as long as an appropriate shape-based restraint for a reference ligand is known. Requiring a docked pose is inherently more time-consuming than the simplest ligand-based models, but the range of applicability of both local Dragnet and ligand-based models is limited, and a relatively small number of ligands are tested. Therefore, computational expense is not generally a practical concern in local Dragnet models. Likewise, as with any end point free energy method, errors incurred by qualitatively inaccurate docking models are inherited by Dragnet. Costly docking models (or explicit Monte Carlo or MD) may greatly exceed the cost of Dragnet itself, at which point more costly free energy approximations may be suitable.

### Transfer Learning

**Figure 5:**
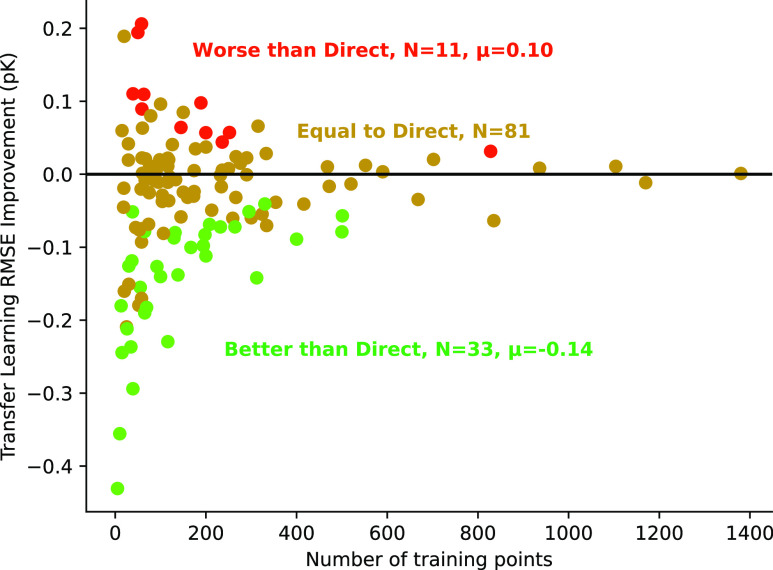
A comparison between transfer-learned Dragnet models and direct-learned models. Each point represents a difference in RMSE between direct and transfer-learned Dragnet models trained on identical fractions of BindingDB local data sets. Green points are data sets where the transfer-learned model is significantly (to within a standard deviation of the model ensemble) better than the direct-learned models. Red points are data sets where transfer-learned models are worse, and yellow points indicate no difference.

We use the global Dragnet model from Figure 3 as a pretrained initialization for transfer-learned models to local data sets. Figure 5 illustrates the performance of transfer-learned models in comparison with direct models. Neural network models trained on little data can exhibit large variance in quality due to overfitting, so we train a 5-member ensemble for each to reduce noise. Importantly, the transfer-learned local Dragnet models are almost always statistically the same or better than direct learning.

We find models on small data sets converge very rapidly with respect to the number of training batches, so the performance is unlikely an artifact from training time. Instead, we believe some useful information is stored in the pretrained weights from the global model, or at least the model is initialized at a more well-behaved region of the loss landscape compared to a random initialization.

**Figure 6:**
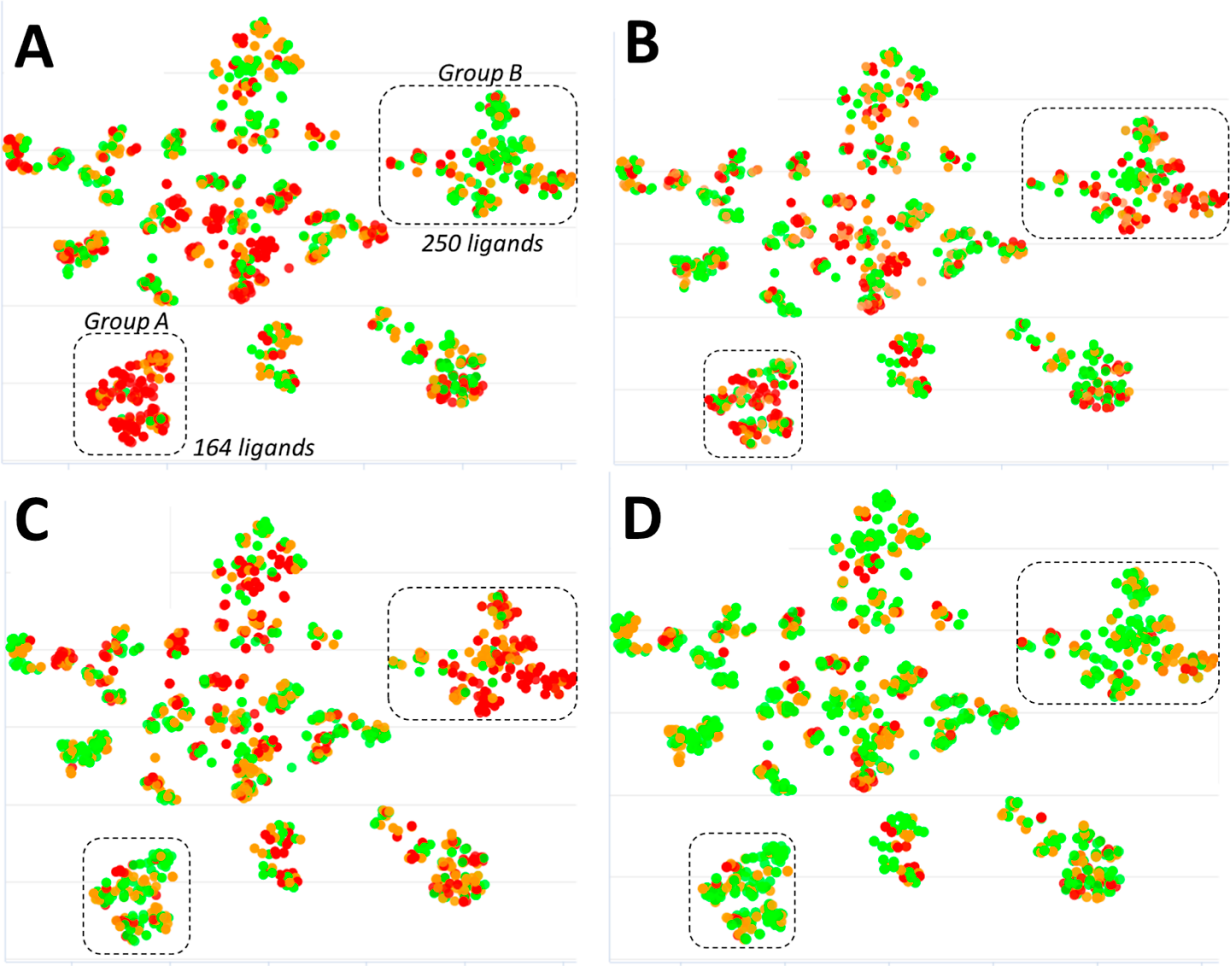
Set 0 test set (ChEMBL S1P_1_ receptor set) chemical space schematic representation for different Dragnet scoring optimization protocols. The chemical space map is based on t-distributed stochastic neighbor embedding (t-SNE) using Morgan fingerprints. Every circle represents a ligand, close circles are similar ligands. Circles are color-coded by the Dragnet prediction error: green are ligands with an error < 1 pEC_50_, orange between 1 and 2, and red above 2 pEC_50_ units. Two clusters are highlighted using dashed boxes: Group A on the bottom left including 164 ligands and Group B on the top right including 250 ligands. (A) Global Dragnet predictions; (B) prediction of global Dragnet optimized via transfer learning using 6 explorer ligands part of Group A with activity predicted using AFEP; (C) prediction of global Dragnet optimized via transfer learning using the same 6 explorer ligands part of Group A using experimental activity; (D) prediction of global Dragnet optimized via transfer learning using 54 diverse explorer ligands using experimental activity.

**Figure 7:**
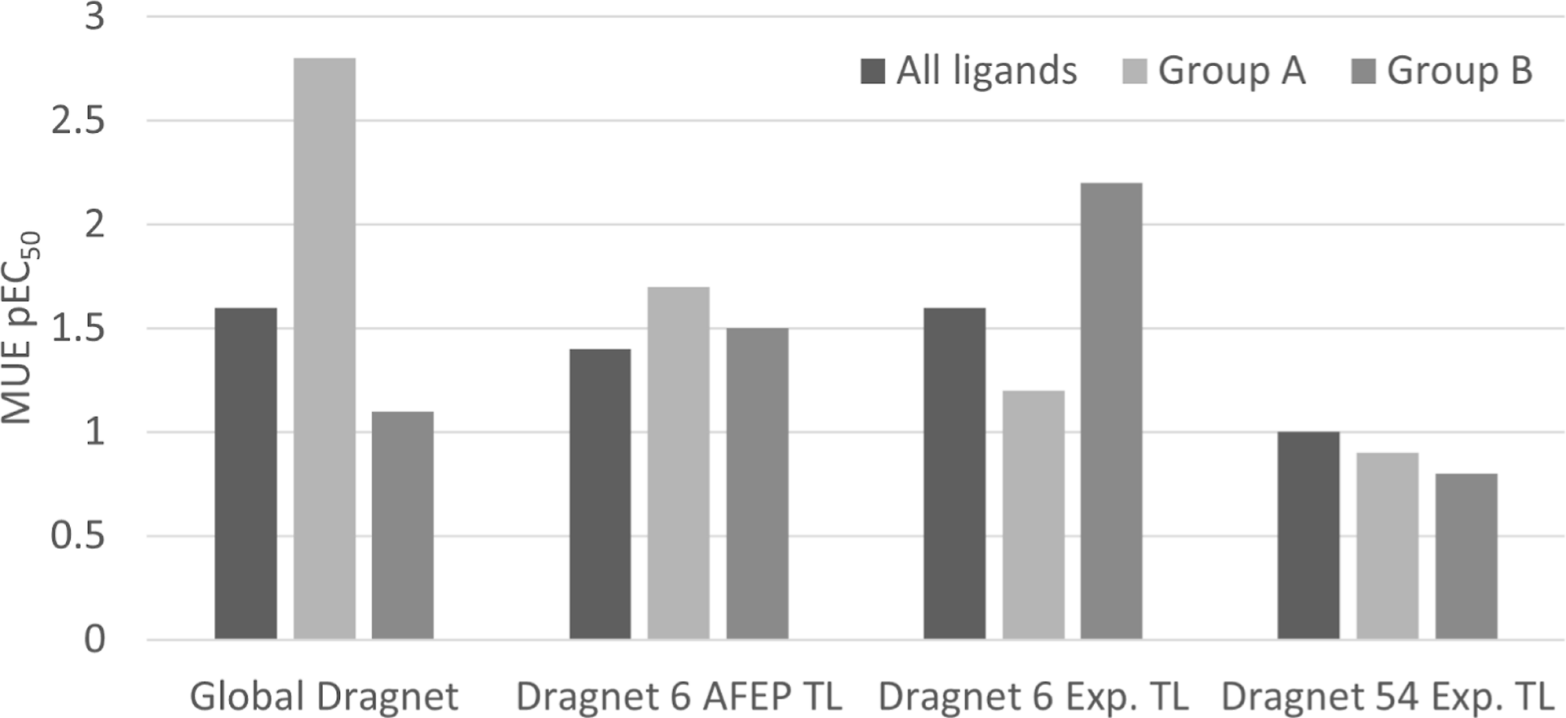
Dragnet MUE in pEC_50_ units for all ligands (black), 164 Group A ligands (light gray), and 250 Group B ligands (dark gray). MUE is reported for the global Dragnet predictions; prediction of global Dragnet optimized via transfer learning using 6 explorer ligands part of Group A with activity predicted using AFEP; prediction of global Dragnet optimized via transfer learning using the same 6 explorer ligands part of Group A using experimental activity; prediction of global Dragnet optimized via transfer learning using 54 diverse explorer ligands using experimental activity.

We further explored the effect of transfer learning on Set 0 which was derived from ChEMBL and includes the functional activity of a diverse set of small molecule agonists of the S1P_1_ receptor. This represents the most challenging case in which Dragnet must infer activation of the GPCR receptor (pEC_50_) from a ligand pose bound to the inactive state of the protein. The global Dragnet scoring function results in a MUE of 1.6 pEC_50_ units, with large variability depending on the chemical scaffold as shown in Figure 6A. A particular chemotype including a pyrimidine (shown as Group A with 164 ligands) was especially challenging, with an MUE reaching 2.8 pEC_50_ (Figure 7). By contrast, the MUE was 1.1 for 250 ligands with an aryl-oxadiazole core (Group B).

Three different transfer learning schemes were evaluated in order to try to better understand the potential value of this strategy. We randomly selected six ligands from Group A (explorer ligands) with the task of obtaining more information about the pyrimidine core chemical space. In the first test (Figure 6B), the binding affinity was predicted using AFEP. The six explorer ligands include four neutral, one negatively charged, and one positively charged compounds, and activity ranging from 6 to 8 pEC_50_. AFEP resulted in MUE = 1.58 pEC_50_ units from experiment: a reasonable performance considering we are using docked poses on an inactive conformation of the receptor and trying to compare predicted binding affinity to functional activity. Transfer learning using these AFEP predictions for the 6 explorer ligands resulted in a more balanced performance of Dragnet (Figure 7) with an improvement of the MUE by 0.2 for all ligands, 0.9 for Group A as opposed to an increase of 0.4 pEC_50_ units for Group B. It is interesting to note that this scoring function’s MUE for Group A ligands is 1.7, which is close to the AFEP MUE for the 6 explorer ligands belonging to that group (1.58 pEC_50_ units). If we use experimental activity, instead of AFEP predictions, for the same 6 ligands, we see a stronger improvement in the MUE for Group A (1.3 pEC_50_), while Group B predictions are negatively affected (MUE reaching 2.2). This suggests that optimizing the scoring function for a particular chemotype can improve the performance with the risk of degradation of the model applicability domain (Figures 6C and 7). To ensure prediction improvements on a broad chemical space, it is necessary to include a set of explorer ligands that is as diverse as the chemical space under consideration. This was tested by expanding the 6 explorers to 54 diverse ligands covering all clusters (Figure 6D). With this transfer learning scheme, Dragnet’s performance was much improved: MUE = 1 pEC_50_ (0.9 for Group A and 0.8 for group B).

## Conclusions

We introduced DrΔ*G*-Net, a modular neural network architecture for protein–ligand binding free energy prediction. We assessed the problems of *global* binding free energy prediction, where one seeks to predict the absolute binding strength of arbitrary protein–ligand pairs, and of *local* binding free energy prediction, where only a congeneric series of ligands are bound to a single protein target. Although these are distinct tasks, they could both be addressed with a GNN. Performance can be enhanced on the latter with a transfer learning procedure. Global Dragnet binding affinity predictive performance was comparable to that of other similar ML-based docking scoring functions. Likewise, for large data sets, prediction accuracy was similar to classic ligand-based ML scoring methods based on 2D fingerprints.

A key, unique feature of Dragnet is the data-efficient optimization via transfer learning using an inherently rotation-invariant neural network readout. A handful of data points defined as exploratory ligands were enough to greatly improve the global scoring function. Local Dragnet can be optimized for a particular scaffold, region of the chemical space, protein target, or different end points. For example, it allowed the use of transfer learning from binding affinity (p*K*_*i*_) to functional activity (pEC_50_). In addition, preliminary results suggest that it is possible to replace experimental data for these explorer ligands with predictions from computationally demanding physics-based methods such as AFEP. Despite the potential errors in these reference methods, the improvement in predictive performance can be remarkable, suggesting Dragnet can extract valuable learning from noisy data, such as docking and AFEP results.

Surprisingly, networks using only a 3-dimensional ligand pose (and no explicit protein information) as input were competitive in the global binding free energy prediction problem. This fact, paired with the convergent performance of disparate modeling strategies, supports the claims of Volkov et al.^14^ that global binding free energy prediction with data-driven methods is unlikely to make significant progress with simple architectural improvements. Dragnet provides a flexible 3D fingerprint framework for ligands alone or in complex with the protein target, which can be used for ML applications. Beyond binding affinity predictions, we can envision cases in which considering the 3D conformation of the ligand can be more effective than a simple 2D representation. For example, considering the conformational changes of a small molecule in solution compared to those in a hydrophobic environment can be relevant to understanding passive cellular permeability.

## Data Availability

The Supporting Information includes all of the requisite data sets (or directions to obtain them), including the pre- and postprocessed complex geometries, MM-GBSA and Glide information, and predictions from our models and the reference 2D ligand-based model. These files are contained at 10.6084/m9.figshare.21354528.v1. Neural network code written for this project is available as a fork of the AP-Net repository at https://github.com/derekmetcalf/apnet and the Mario pipeline is available at https://github.com/andyj10224/mario.

## Supporting Information Available

Details about specific hyperparameter choices, architectural details, and training procedures for neural networks (PDF)

## References

[ref1] Takenaka T. Classical Vs Reverse Pharmacology in Drug Discovery. BJU Int. 2001, 88, 7–10. 10.1111/j.1464-410X.2001.00112.x. 11589663

[ref2] Lee J. A.; Uhlik M. T.; Moxham C. M.; Tomandl D.; Sall D. J. Modern Phenotypic Drug Discovery is a Viable, Neoclassic Pharma Strategy. J. Med. Chem. 2012, 55, 4527–4538. 10.1021/jm201649s. 22409666

[ref3] Hughes J. P.; Rees S. S.; Kalindjian S. B.; Philpott K. L. Principles of Early Drug Discovery. Br. J. Pharmacol. 2011, 162, 1239–1249. 10.1111/j.1476-5381.2010.01127.x. 21091654 PMC3058157

[ref4] Aminpour M.; Montemagno C.; Tuszynski J. A. An Overview of Molecular Modeling for Drug Discovery with Specific Illustrative Examples of Applications. Molecules 2019, 24, 1693 10.3390/molecules24091693. 31052253 PMC6539951

[ref5] Zhao L.; Ciallella H. L.; Aleksunes L. M.; Zhu H. Advancing Computer-Aided Drug Discovery (CADD) by Big Data and Data-Driven Machine Learning Modeling. Drug Discovery Today 2020, 25, 1624–1638. 10.1016/j.drudis.2020.07.005. 32663517 PMC7572559

[ref6] Cournia Z.; Allen B.; Sherman W. Relative Binding Free Energy Calculations in Drug Discovery: Recent Advances and Practical Considerations. J. Chem. Inf. Model. 2017, 57, 2911–2937. 10.1021/acs.jcim.7b00564. 29243483

[ref7] Cournia Z.; Allen B. K.; Beuming T.; Pearlman D. A.; Radak B. K.; Sherman W. Rigorous Free Energy Simulations in Virtual Screening. J. Chem. Inf. Model. 2020, 60, 4153–4169. 10.1021/acs.jcim.0c00116. 32539386

[ref8] Mey A. S. J. S.; Allen B. K.; Bruce Macdonald H. E.; Chodera J. D.; Hahn D. F.; Kuhn M.; Michel J.; Mobley D. L.; Naden L. N.; Prasad S.; Rizzi A.; Scheen J.; Shirts M. R.; Tresadern G.; Xu H. Best Practices for Alchemical Free Energy Calculations [article V1.0]. Living J. Comp. Mol. Sci. 2020, 2, 18378 10.33011/livecoms.2.1.18378. PMC8388617 34458687

[ref9] Hahn D.; Bayly C.; Boby M. L.; Bruce Macdonald H.; Chodera J.; Gapsys V.; Mey A.; Mobley D.; Benito L. P.; Schindler C.; Tresadern G.; Warren G. Best Practices for Constructing, Preparing, and Evaluating Protein-Ligand Binding Affinity Benchmarks [article V1.0]. Living J. Comp. Mol. Sci. 2022, 4, 1497 10.33011/livecoms.4.1.1497. PMC9662604 36382113

[ref10] Guvench O.; MacKerell A. D. Jr. Computational Fragment-Based Binding Site Identification by Ligand Competitive Saturation. PLoS Comput. Biol. 2009, 5, 10004355 10.1371/journal.pcbi.1000435. PMC2700966 19593374

[ref11] Faller C. E.; Raman E. P.; MacKerell A. D.; Guvench O. In Fragment-Based Methods in Drug Discovery; Klon A. E., Ed.; Springer New York: New York, NY, 2015; pp 75–87. 10.1007/978-1-4939-2486-8_7 PMC4685950 25709034

[ref12] Prime; Schrödinger; LLC: New York, NY, 2021.

[ref13] Wang E.; Sun H.; Wang J.; Wang Z.; Liu H.; Zhang J. Z.; Hou T. End-Point Binding Free Energy Calculation with MM/PBSA and MM/GBSA: Strategies and Applications in Drug Design. Chem. Rev. 2019, 119, 9478–9508. 10.1021/acs.chemrev.9b00055. 31244000

[ref14] Volkov M.; Turk J.-A.; Drizard N.; Martin N.; Hoffmann B.; Gaston-Mathé Y.; Rognan D. On The Frustration to Predict Binding Affinities from Protein–ligand Structures with Deep Neural Networks. J. Med. Chem. 2022, 65, 7946–7958. 10.1021/acs.jmedchem.2c00487. 35608179

[ref15] Meli R.; Morris G. M.; Biggin P. C. Scoring Functions for Protein-Ligand Binding Affinity Prediction using Structure-Based Deep Learning: A Review. Front. Bioinf. 2022, 2, 57 10.3389/fbinf.2022.885983. PMC7613667 36187180

[ref16] Thomas M.; et al. Comparison of Structure- and Ligand-Based Scoring Functions for Deep Generative Models: A GPCR Case Study. J. Cheminf. 2021, 13, 39 10.1186/s13321-021-00516-0. PMC8117600 33985583

[ref17] Zagidullin B.; Wang Z.; Guan Y.; Pitkänen E.; Tang J. Comparative Analysis of Molecular Fingerprints in Prediction of Drug Combination Effects. Briefings Bioinf. 2021, 22, bbab291 10.1093/bib/bbab291. PMC8574997 34401895

[ref18] Torng W.; Altman R. B. Graph Convolutional Neural Networks for Predicting Drug-Target Interactions. J. Chem. Inf. Model. 2019, 59, 4131–4149. 10.1021/acs.jcim.9b00628. 31580672

[ref19] von Korff M.; Sander T. Limits of Prediction for Machine Learning in Drug Discovery. Front. Pharmacol 2022, 13, 588 10.3389/fphar.2022.832120. PMC8960959 35359835

[ref20] Bortolato A.; Fanton M.; Mason J. S.; Moro S. In Biomolecular Simulations: Methods and Protocols; Monticelli L., Salonen E., Eds.; Humana Press: Totowa, NJ, 2013; pp 339–360.

[ref21] Velec H. F.; Gohlke H.; Klebe G. DrugScore^CSD^ Knowledge-Based Scoring Function Derived from Small Molecule Crystal Data with Superior Recognition Rate of Near-Native Ligand Poses and Better Affinity Prediction. J. Med. Chem. 2005, 48, 6296–6303. 10.1021/jm050436v. 16190756

[ref22] Ballester P. J.; Mitchell J. B. A Machine Learning Approach to Predicting Protein–Ligand Binding Affinity with Applications to Molecular Docking. Bioinformatics 2010, 26, 1169–1175. 10.1093/bioinformatics/btq112. 20236947 PMC3524828

[ref23] Gilmer J.; Schoenholz S. S.; Riley P. F.; Vinyals O.; Dahl G. E. Neural Message Passing for Quantum Chemistry. arXiv 2017, arXiv:1704.01212.

[ref24] Öztürk H.; Özgür A.; Ozkirimli E. D.D. T. A. DeepDTA: deep drug–target binding affinity prediction. Bioinformatics 2018, 34, i821–i829. 10.1093/bioinformatics/bty593. 30423097 PMC6129291

[ref25] Karimi M.; Wu D.; Wang Z.; Shen Y. DeepAffinity: Interpretable Deep Learning of Compound–Protein Affinity Through Unified Recurrent and Convolutional Neural Networks. Bioinformatics 2019, 35, 3329–3338. 10.1093/bioinformatics/btz111. 30768156 PMC6748780

[ref26] Li H.; Sze K.-H.; Lu G.; Ballester P. J. Machine-Learning Scoring Functions for Structure-Based Drug Lead Optimization. Wiley Interdiscip. Rev.: Comput. Mol. Sci. 2020, 10, e1465 10.1002/wcms.1465. PMC4832270 27110292

[ref27] Shen C.; Ding J.; Wang Z.; Cao D.; Ding X.; Hou T. From Machine Learning to Deep Learning: Advances in Scoring Functions for Protein–Ligand Docking. Wiley Interdiscip. Rev.: Comput. Mol. Sci. 2020, 10, e1429 10.1002/wcms.1429.

[ref28] Meng Z.; Xia K. Persistent Spectral-Based Machine Learning (Perspect ML) for Protein-Ligand Binding Affinity Prediction. Sci. Adv. 2021, 7, eabc5329 10.1126/sciadv.abc5329. 33962954 PMC8104863

[ref29] Li H.; Peng J.; Sidorov P.; Leung Y.; Leung K.-S.; Wong M.-H.; Lu G.; Ballester P. J. Classical Scoring Functions for Docking are Unable to Exploit Large Volumes of Structural and Interaction Data. Bioinformatics 2019, 35, 3989–3995. 10.1093/bioinformatics/btz183. 30873528

[ref30] Sánchez-Cruz N.; Medina-Franco J. L.; Mestres J.; Barril X. Extended Connectivity Interaction Features: Improving Binding Affinity Prediction Through Chemical Description. Bioinformatics 2021, 37, 1376–1382. 10.1093/bioinformatics/btaa982. 33226061

[ref31] Feinberg E. N.; Sur D.; Wu Z.; Husic B. E.; Mai H.; Li Y.; Sun S.; Yang J.; Ramsundar B.; Pande V. S. Potentialnet for Molecular Property Prediction. ACS Central Sci. 2018, 4, 1520–1530. 10.1021/acscentsci.8b00507. PMC6276035 30555904

[ref32] Jiang M.; Li Z.; Zhang S.; Wang S.; Wang X.; Yuan Q.; Wei Z. Drug–Target Affinity Prediction using Graph Neural Network and Contact Maps. RSC Adv. 2020, 10, 20701–20712. 10.1039/D0RA02297G. 35517730 PMC9054320

[ref33] Knutson C.; Bontha M.; Bilbrey J. A.; Kumar N. Decoding the Protein–ligand Interactions using Parallel Graph Neural Networks. Sci. Rep. 2022, 12, 7624–7714. 10.1038/s41598-022-10418-2. 35538084 PMC9086424

[ref34] Unke O. T.; Meuwly M. PhysNet: A Neural Network for Predicting Energies, Forces, Dipole Moments, and Partial Charges. J. Chem. Theory Comput. 2019, 15, 3678–3693. 10.1021/acs.jctc.9b00181. 31042390

[ref35] Ko T. W.; Finkler J. A.; Goedecker S.; Behler J. A Fourth-Generation High-Dimensional Neural Network Potential with Accurate Electrostatics Including Non-Local Charge Transfer. Nat. Commun. 2021, 12, 398 10.1038/s41467-020-20427-2. 33452239 PMC7811002

[ref36] Satorras V. G.; Hoogeboom E.; Welling M. E(n) Equivariant Graph Neural Networks. arXiv 2021, arXiv:2102.09844.

[ref37] Batzner S.; Musaelian A.; Sun L.; Geiger M.; Mailoa J. P.; Kornbluth M.; Molinari N.; Smidt T. E.; Kozinsky B. E(3)-Equivariant Graph Neural Networks for Data-Efficient and Accurate Interatomic Potentials. arXiv 2021, arXiv:2101.03164. 10.1038/s41467-022-29939-5 PMC9068614 35508450

[ref38] Schütt K. T.; Kindermans P.-J.; Sauceda H. E.; Chmiela S.; Tkatchenko A.; Müller K.-R. SchNet: A Continuous-Filter Convolutional Neural Network for Modeling Quantum Interactions. arXiv 2017, arXiv:1706.08566.

[ref39] Musaelian A.; Batzner S.; Johansson A.; Sun L.; Owen C. J.; Kornbluth M.; Kozinsky B. Learning Local Equivariant Representations for Large-Scale Atomistic Dynamics. arXiv 2022, arXiv:2204.05249. 10.1038/s41467-023-36329-y PMC9898554 36737620

[ref40] Behler J.; Parrinello M. Generalized Neural-Network Representation of High-Dimensional Potential-Energy Surfaces. Phys. Rev. Lett. 2007, 98, 146401 10.1103/PhysRevLett.98.146401. 17501293

[ref41] Glick Z. L.; Metcalf D. P.; Koutsoukas A.; Spronk S. A.; Cheney D. L.; Sherrill C. D. AP-Net: An Atomic-Pairwise Neural Network for Smooth and Transferable Interaction Potentials. J. Chem. Phys. 2020, 153, 044112 10.1063/5.0011521. 32752707

[ref42] Metcalf D. P.; Koutsoukas A.; Spronk S. A.; Claus B. L.; Loughney D. A.; Johnson S. R.; Cheney D. L.; Sherrill C. D. Approaches for Machine Learning Intermolecular Interaction Energies and Application to Energy Components from Symmetry Adapted Perturbation Theory. J. Chem. Phys. 2020, 152, 074103 10.1063/1.5142636. 32087645

[ref43] Smith J. S.; Isayev O.; Roitberg A. E. ANI-1: An Extensible Neural Network Potential with DFT Accuracy at Force Field Computational Cost. Chem. Sci. 2017, 8, 3192–3203. 10.1039/C6SC05720A. 28507695 PMC5414547

[ref44] Smith J. S.; Nebgen B. T.; Zubatyuk R.; Lubbers N.; Devereux C.; Barros K.; Tretiak S.; Isayev O.; Roitberg A. E. Approaching Coupled Cluster Accuracy with A General-Purpose Neural Network Potential through Transfer Learning. Nat. Commun. 2019, 10, 2903 10.1038/s41467-019-10827-4. 31263102 PMC6602931

[ref45] Yao K.; Herr J. E.; Toth D.; Mckintyre R.; Parkhill J. The Tensormol-0.1 Model Chemistry: A Neural Network Augmented with Long-Range Physics. Chem. Sci. 2018, 9, 2261–2269. 10.1039/C7SC04934J. 29719699 PMC5897848

[ref46] Glick Z. L.; Metcalf D. P.; Sargent C. T.; Spronk S. A.; Koutsoukas A.; Cheney D. L.; Sherrill C. D. A Physics-Aware Neural Network for Protein-Ligand Interactions with Quantum Chemical Accuracy. ChemRxiv 2024, 10.26434/chemrxiv-2024-5v6gh. PMC11339967 39183910

[ref47] Glick Z. L. AP-net GitHub Repository. https://github.com/zachglick/apnet, 2023.

[ref48] Boyles F.; Deane C. M.; Morris G. M. Learning from the ligand: Using Ligand-Based Features to Improve Binding Affinity Prediction. Bioinformatics 2020, 36, 758–764. 10.1093/bioinformatics/btz665. 31598630

[ref49] Wang R.; Fang X.; Lu Y.; Yang C. Y.; Wang S. The PDBBind Database: Methodologies and Updates. J. Med. Chem. 2005, 48, 4111–4119. 10.1021/jm048957q. 15943484

[ref50] Liu Z.; Su M.; Han L.; Liu J.; Yang Q.; Li Y.; Wang R. Forging The Basis for Developing Protein-Ligand Interaction Scoring Functions. Acc. Chem. Res. 2017, 50, 302–309. 10.1021/acs.accounts.6b00491. 28182403

[ref51] Su M.; Yang Q.; Du Y.; Feng G.; Liu Z.; Li Y.; Wang R. Comparative Assessment of Scoring Functions: The CASF-2016 Update. J. Chem. Inf. Model. 2019, 59, 895–913. 10.1021/acs.jcim.8b00545. 30481020

[ref52] Gilson M. K.; Liu T.; Baitaluk M.; Nicola G.; Hwang L.; Chong J. BindingDB in 2015: A Public Database for Medicinal Chemistry, Computational Chemistry and Systems Pharmacology. Nucleic Acids Res. 2016, 44, D1045–D1053. 10.1093/nar/gkv1072. 26481362 PMC4702793

[ref53] Davies M.; Nowotka M.; Papadatos G.; Dedman N.; Gaulton A.; Atkinson F.; Bellis L.; Overington J. P. ChEMBL Web Services: Streamlining Access to Drug Discovery Data and Utilities. Nucleic Acids Res. 2015, 43, W612–W620. 10.1093/nar/gkv352. 25883136 PMC4489243

[ref54] Maestro; Schrödinger LLC: New York, NY, 2021.

[ref55] Protein Preparation Wizard; Epik, Schrödinger, LLC, New York, NY, 2021.

[ref56] Impact; Schrödinger LLC, New York, NY.

[ref57] Friesner R. A.; Banks J. L.; Murphy R. B.; Halgren T. A.; Klicic J. J.; Mainz D. T.; Repasky M. P.; Knoll E. H.; Shelley M.; Perry J. K.; Shaw D. E.; Francis P.; Shenkin P. S. Glide: A New Approach for Rapid, Accurate Docking and Scoring. 1. Method and Assessment of Docking Accuracy. J. Med. Chem. 2004, 47, 1739–1749. 10.1021/jm0306430. 15027865

[ref58] Hanson M. A.; Roth C. B.; Jo E.; Griffith M. T.; Scott F. L.; Reinhart G.; Desale H.; Clemons B.; Cahalan S. M.; Schuerer S. C.; Sanna M. G.; Han G. W.; Kuhn P.; Rosen H.; Stevens R. C. Crystal Structure of a Lipid G Protein–Coupled Receptor. Science 2012, 335, 851–855. 10.1126/science.1215904. 22344443 PMC3338336

[ref59] Jacobson M. P.; Pincus D. L.; Rapp C. S.; Day T. J.; Honig B.; Shaw D. E.; Friesner R. A. A Hierarchical Approach to All-Atom Protein Loop Prediction. Proteins: Struct., Funct., Genet. 2004, 55, 351–367. 10.1002/prot.10613. 15048827

[ref60] Sanna M. G.; Wang S.-K.; Gonzalez-Cabrera P. J.; Don A.; Marsolais D.; Matheu M. P.; Wei S. H.; Parker I.; Jo E.; Cheng W.-C.; Cahalan M. D.; Wong C.-H.; Rosen H. Enhancement of Capillary Leakage and Restoration of Lymphocyte Egress by a Chiral S1P1 Antagonist in Vivo. Nat. Chem. Bio. 2006, 2, 434–441. 10.1038/nchembio804. 16829954

[ref61] Desmond Molecular Dynamics System; D.E. Shaw Research: New York, NY, USA.

[ref62] Chen W.; Cui D.; Abel R.; Friesner R. A.; Wang L. Accurate Calculation of Absolute Protein-Ligand Binding Free Energies. ChemRxiv 2022, 10.26434/chemrxiv-2022-2t0dq-v2.

[ref63] Jiménez-Luna J.; Pérez-Benito L.; Martínez-Rosell G.; Sciabola S.; Torella R.; Tresadern G.; De Fabritiis G. DeltaDelta Neural Networks for Lead Optimization of Small Molecule Potency. Chem. Sci. 2019, 10, 10911–10918. 10.1039/C9SC04606B. 32190246 PMC7066671

[ref64] Wang C.; Zhang Y. Improving Scoring-Docking-Screening Powers of Protein–Ligand Scoring Functions using Random Forest. J. Comput. Chem. 2017, 38, 169–177. 10.1002/jcc.24667. 27859414 PMC5140681

[ref65] Trott O.; Olson A. J. Autodock Vina: Improving The Speed and Accuracy of Docking with A New Scoring Function, Efficient Optimization, and Multithreading. J. Comput. Chem. 2010, 31, 455–461. 10.1002/jcc.21334. 19499576 PMC3041641

[ref66] Nguyen D. D.; Wei G.-W. AGL-Score: Algebraic Graph Learning Score for Protein-Ligand Binding Scoring, Ranking, Docking, and Screening. J. Chem. Inf. Model. 2019, 59, 3291–3304. 10.1021/acs.jcim.9b00334. 31257871 PMC6664294

[ref67] Cang Z.; Mu L.; Wei G.-W. Representability of Algebraic Topology for Biomolecules in Machine Learning Based Scoring and Virtual Screening. PLoS Comput. Biol. 2018, 14, e1005929 10.1371/journal.pcbi.1005929. 29309403 PMC5774846

[ref68] Liu X.; Feng H.; Wu J.; Xia K. Persistent Spectral Hypergraph Based Machine Learning (PSH-ML) for Protein-Ligand Binding Affinity Prediction. Briefings Bioinf. 2021, 22, bbab127 10.1093/bib/bbab127. 33837771

[ref69] Halgren T. A.; Murphy R. B.; Friesner R. A.; Beard H. S.; Frye L. L.; Pollard W. T.; Banks J. L. Glide: A New Approach for Rapid, Accurate Docking and Scoring. 2. Enrichment Factors in Database Screening. J. Med. Chem. 2004, 47, 1750–1759. 10.1021/jm030644s. 15027866

[ref70] Friedman J. H. Greedy Function Approximation: A Gradient Boosting Machine. Ann. Stat. 2001, 29, 1189–1232. 10.1214/aos/1013203451.

[ref71] Morgan H. L. The Generation of a Unique Machine Description for Chemical Structures—A Technique Developed at Chemical Abstracts Service. J. Chem. Doc. 1965, 5, 107–113. 10.1021/c160017a018.

