# Supplementary material for: Directional ΔG Neural Network (DrΔG-Net): A Modular Neural Network Approach to Binding Free Energy Prediction
Source: J Chem Inf Model. 2024 Mar 12;64(6):1907–18. doi: 10.1021/acs.jcim.3c02054 (PMC10966643; doi:10.1021/acs.jcim.3c02054)
Supplement: Supplementary file 1 — ci3c02054_si_001.pdf [file ci3c02054_si_001.pdf]

Supporting Information for Directional ΔG Neural Network (DrΔG-Net): A modular neural network approach to binding free energy prediction

Derek P. Metcalf,[†] Zachary L. Glick,[†] Andrea Bortolato,[‡] Andy Jiang,[†] Daniel L. Cheney,[‡] and C. David Sherrill[*,†]

[†]*Center for Computational Molecular Science and Technology, School of Chemistry and Biochemistry and School of Computational Science and Engineering, Georgia Institute of Technology, Atlanta, Georgia 30332-0400, USA*

[‡]*Molecular Structure and Design, Bristol-Myers Squibb Company, P. O. Box 5400, Princeton, NJ 08543*

E-mail: sherrill@gatech.edu.

Neural Network Architectural Details

The Dragnet model is written in the neural network library Tensorflow 2.3. All neural network widths and depths used for this study are the defaults in the Dragnet program. SeLU activation functions are used throughout. The cutoff function for the readout and sparse message passing is a piecewise cosine decay that goes to zero at 5Å. The readout function scaling constants c_L and c_{PL} from are initialized to 10^{-5} and 10^{-7}, respectively, and are allowed to change during training, but in practice remain near their initial values.

Neural Network Training Details

All experiments utilize 5-fold cross validation to determine a set of optimal hyperparameters. Dropout is shown to improve or retain performance in all experiments and a value of 0.1 is chosen for all weights. Likewise, data augmentation in the form of injecting random Cartesian noise (0.1Å) to nuclei improves performance and is used for all experiments. We use the Adam optimizer, clipping gradient norms to 0.05 and we use a cosine decay warmup schedule that begins at learning rate 0.0 and reaches peak learning rate after one epoch. Transfer learning experiments simply utilize warm starts rather than freeze any layer weights, as this gave the best performance. In "local" experiments, which generally have much less data, we initialize a learning rate of 10^{-6}, whether transfer learning or not, whereas in "global" experiments, we initialize a learning rate of 5×10^{-5}. All models are trained for 500 epochs or until the validation error has not increased for 50 epochs. In our experiments, the latter condition is always reached before the former and convergence is achieved.
